# Modified coptisine derivatives as an inhibitor against pathogenic *Rhizomucor miehei*, *Mycolicibacterium smegmatis* (Black Fungus), Monkeypox, and Marburg virus by molecular docking and molecular dynamics simulation-based drug design approach

**DOI:** 10.3389/fphar.2023.1140494

**Published:** 2023-04-19

**Authors:** Shopnil Akash, Arafat Hossain, Nobendu Mukerjee, Md. Moklesur Rahman Sarker, Mohammad Firoz Khan, Md. Jamal Hossain, Mohammad A. Rashid, Ajoy Kumer, Arabinda Ghosh, Darwin A. León-Figueroa, Joshuan J. Barboza, Bijaya Kumar Padhi, Ranjit Sah

**Affiliations:** ^1^ Department of Pharmacy, Faculty of Allied Health Sciences, Daffodil International University, Dhaka, Bangladesh; ^2^ Department of Biochemistry and Molecular Biology, Bangabandhu Sheikh Mujibur Rahman Science and Technology University, Gopalganj, Bangladesh; ^3^ Department of Microbiology, West Bengal State University, Kolkata, West Bengal, India; ^4^ Department of Health Sciences, Novel Global Community Educational Foundation, Hebersham, NSW, Australia; ^5^ Health Med. Science Research Network, Dhaka, Bangladesh; ^6^ Department of Pharmacy, State University of Bangladesh, Dhaka, Bangladesh; ^7^ Department of Pharmaceutical Chemistry, Faculty of Pharmacy, University of Dhaka, Dhaka, Bangladesh; ^8^ Laboratory of Computational Research for Drug Design and Material Science, Department of Chemistry, European University of Bangladesh, Dhaka, Bangladesh; ^9^ Microbiology Division, Department of Botany, Gauhati University, Guwahati, Assam, India; ^10^ Facultad de Medicina Humana, Universidad de San Martín de Porres, Chiclayo, Peru; ^11^ Escuela de Medicina, Universidad Cesar Vallejo, Trujillo, Peru; ^12^ Department of Community Medicine, School of Public Health, Postgraduate Institute of Medical Education and Research, Chandigarh, India; ^13^ Institute of Medicine, Tribhuvan University Teaching Hospital, Kathmandu, Nepal; ^14^ Dr. D.Y Patil Medical College, Hospital and Research Centre, Pune, Maharashtra, India; ^15^ Green City Hospital, Tokha, Nepal

**Keywords:** Black Fungus, Monkeypox, Marburg virus, molecular docking, admet, QSAR, molecular dynamic simulation, DFT

## Abstract

During the second phase of SARS-CoV-2, an unknown fungal infection, identified as black fungus, was transmitted to numerous people among the hospitalized COVID-19 patients and increased the death rate. The black fungus is associated with the Mycolicibacterium smegmatis, Mucor lusitanicus, and Rhizomucor miehei microorganisms. At the same time, other pathogenic diseases, such as the Monkeypox virus and Marburg virus, impacted global health. Policymakers are concerned about these pathogens due to their severe pathogenic capabilities and rapid spread. However, no standard therapies are available to manage and treat those conditions. Since the coptisine has significant antimicrobial, antiviral, and antifungal properties; therefore, the current investigation has been designed by modifying coptisine to identify an effective drug molecule against Black fungus, Monkeypox, and Marburg virus. After designing the derivatives of coptisine, they have been optimized to get a stable molecular structure. These ligands were then subjected to molecular docking study against two vital proteins obtained from black fungal pathogens: Rhizomucor miehei (PDB ID: 4WTP) and Mycolicibacterium smegmatis (PDB ID 7D6X), and proteins found in Monkeypox virus (PDB ID: 4QWO) and Marburg virus (PDB ID 4OR8). Following molecular docking, other computational investigations, such as ADMET, QSAR, drug-likeness, quantum calculation and molecular dynamics, were also performed to determine their potentiality as antifungal and antiviral inhibitors. The docking score reported that they have strong affinities against Black fungus, Monkeypox virus, and Marburg virus. Then, the molecular dynamic simulation was conducted to determine their stability and durability in the physiological system with water at 100 ns, which documented that the mentioned drugs were stable over the simulated time. Thus, our *in silico* investigation provides a preliminary report that coptisine derivatives are safe and potentially effective against Black fungus, Monkeypox virus, and Marburg virus. Hence, coptisine derivatives may be a prospective candidate for developing drugs against Black fungus, Monkeypox and Marburg viruses.

## Introduction

The SARS-CoV-2 infection, which is responsible for the ongoing disease outbreak and has caused a rise in the percentage of COVID instances with the existing new wave, is a significant concern in terms of global health, particularly for immunocompromised individuals and elderly people. Ebola, Zika, Influenza, SARS-CoV, MERS-CoV-2, Monkeypox and Marburg are just some of the viral infections that have infected millions of humans, animals, and birds alike as either a seasonal epidemic, a pandemic, or a global health emergency (Mohapatra et al., 2021). In the post COVID-19 era or second phase of SARS-CoV-2, a deadly fungal pathogen identified as black fungus (particularly the species of *Mucormycosis*) infected a large number of SARS-CoV-2 affected patients in Indian and Bangladesh subcontinents.

When mucormycosis infects patients, they form black abnormalities in color; this is one of the reasons mucormycosis is sometimes referred to as “black fungus.” Mucormycosis is mainly a group of molds containing filaments that belong to the mucoromycetes. This particular kind of pathogenic fungus grows almost exclusively on rotting vegetables, bread, dirt, and dust. Humans interact with these pathogens by breathing spores, consuming food that has been compromised, or inoculating exposed skin or sores. Scientists have discovered that black fungus may damage the primary organs of the human body, such as the liver, kidney, *etc.* Based on findings from the last two decades, mucormycosis has emerged as a scary fungal illness with elevated death rates among all other fungi infections (Dubey et al., 2021).

Amphotericin B, echinocandins, flucytosine, and azoles are the most commonly prescribed medications for infections caused by the fungus, and these categories of antifungal medications offer different mechanisms of action. Among the most effective forms of antifungal drugs, azole antifungals are considered first-line treatments for fungal infections because of their high activity level across a broad range of fungal strains and their systemic availability. The great concern now is the global resistance to antifungal drugs such as fluconazole-resistant *Candida albicans* (Marchaim et al., 2012), amphotericin B, and fluconazole-resistant *Candida auris* (Sarma et al., 2013). In addition, numerous antifungal medications have been documented to have adverse effects on host tissue. While azoles are often used to treat Aspergillosis, hepatotoxicity and visual disturbances have been reported as adverse effects. Besides, no established medication is available in the market for the treatment of black fungus.

Simultaneously, two more pathogenic viral infections have recently affected numerous people, which have occurred due to the Monkeypox and Marburg viruses. The spread of these two viruses might trigger another global health emergency and may turn into a global health crisis. Monkeypox is indigenous to West and Central Africa and is infected by a virus classified in the same clade as smallpox and cowpox (MacNeil et al., 2009). Historically, Monkeypox infections have been documented mostly in Central Africa, with the first occurrence confirmed in the Democratic Republic of Congo (DRC) in 1970. Human infections with the Monkeypox virus were infrequent outside Africa until April 2022. But recently, it has been happening all across the globe, and it is not clear how infections spread, what variables put people at risk, how the disease manifests in the body, or what the consequences of infection are. According to a few studies, the Monkeypox virus is transmitted from person to person through direct animal contact (Quarleri et al., 2022). The clinical features of MPXV are comparable to those caused by the smallpox virus. MPXV is categorized as a member of the genus Orthopoxvirus and has a double-stranded DNA genome. Comparatively, the MPXV genome has around 190 genes, while the orthopoxvirus genome contains approximately 200 genes (Wang et al., 2022). The symptoms of Monkeypox include a high temperature (38.5^°^ to 40.5^°^C), fatigue, a rash, and a headache. Lymph node enlargement and the presence of hard, deep, well-circumscribed, umbilicated lesions are very suggestive (Duque et al., 2022).

Secondly, the Marburg virus, often known as MARV, is a zoonotic pathogen that may spread from animals to people and produce epidemics of severe infection. It is considered a member of a distinct genus and is classed as the filovirus family, which also includes the Ebola virus (EBOV). It is an encapsulated, negative-sense RNA virus, which may produce epidemics of a severe, sometimes deadly disease in people (Zhao et al., 2022). Since its discovery in 1967, the Marburg virus (MARV) has been a significant cause of worry, with two crucial outbreaks occurring between 1998 and 2004 (Abir et al., 2022). In the Ashanti area of Ghana, there have been two confirmed fatal instances of the Marburg virus disease (MVD). The appropriate health authorities notified these patients of possible viral hemorrhagic fever (VHF) on 28 June 2022, and on 1 July 2022, they tested positive for the Marburg virus (Sah et al., 2022). Although these pathogenic infections are continuously happening, an effective treatment to manage them is lacking. Consequently, this urgently necessitates the development of new antifungal and antiviral drugs with more potent antifungal and antiviral effectiveness with fewer adverse effects.

Coptisine is a natural alkaloid that may be detected in Chinese goldthread. This alkaloid is used in traditional Chinese herbal medicine to treat digestion problems caused by pathogenic bacteria (Gobato et al., 2015). It has a wide variety of medicinal benefits, such as antidiabetic, antimicrobial, antiviral, anti-cancer (Wang et al., 2017). So, for the convenience of this research investigation, coptisine has been modified in addition to different functional groups to determine the probable antifungal, and antiviral efficacy and effectiveness. The synthesis of efficient medication in current medical research is a sophisticated process that calls for a substantial amount of resources, time, money, and human labor. As a direct consequence of this, the use of *in silico* methodologies has developed into a major tenet in the process of the discovery of novel bioactive compounds (Mohapatra et al., 2020). Their significance in drug development is that they are most effective in uncovering and exploring different potential drugs while minimizing costs and time.

## Computational method and working procedure

### Modified structure design of coptisine

**FIGURE 1:**
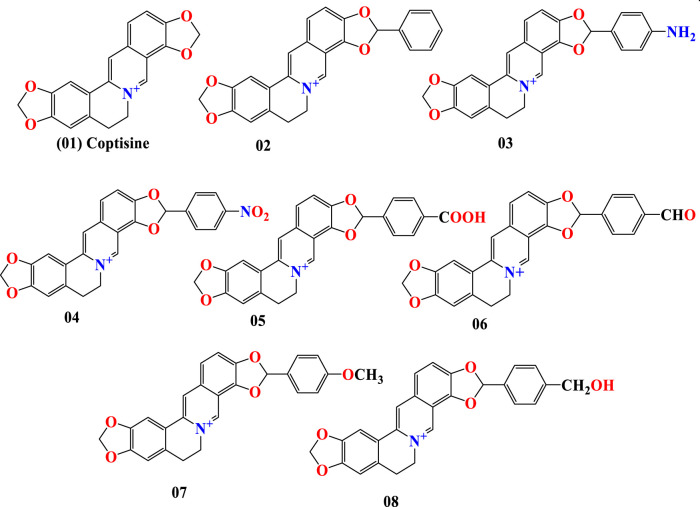
Chemical structure of coptisine and its derivatives.

Coptisine is a conventional quaternary alkaloid generated from the benzylisoquinolines through phenolic oxidation. Its chemical formula is C_19_H_14_NO_4_, and it is synthesized from benzylisoquinolines by phenolic oxidation and bonding with the isoquinoline N-methyl group (Macáková et al., 2019). Structure-based drug discovery comprises creating and improving chemical structures to locate a potentially beneficial curative candidate for laboratory trials (Andricopulo et al., 2009). Following the development of the first possible lead chemical, optimization work has been carried out to ensure an efficient therapeutic candidate. It depends on a grasp of the three-dimensional structure of the substance and how its shape, properties, and charge cause it to engage with the target organism. It is also intended to treat the leads to the production of pharmacologically efficacies molecules. So, in this investigation, the primary molecule was coptisine, which was modified (01–08) in addition to different functional groups. The modifications are depicted in Figure 1.

Since aromatic rings in compounds have a higher degree of freedom, they are able to engage more strongly with particular protein target. Because of presence in aromatic parts, they play a role in protein stability, and they are also thought to play a role in the enhancement of affinity and specificity in drug-like compounds, making them an important component in the development of therapeutics (Lanzarotti et al., 2020; Azam et al., 2021).

### Optimization and ligand preparation

For geometry optimization, the Material Studio 08 version has been applied using B3LYP and the functional unit DFT procedure of DMoL3 code. This technique has been involved to achieve accurate results. Due to the existence of the electronegative atom, oxygen, the B3LYP functional and basis set DND was appropriately arranged. Following the completion of geometric optimization, the optimized lead compounds were saved as pdb files for further computational research, such as molecular docking, molecular dynamic simulation, and ADMET analysis (Kumer et al., 2022a). The Frontier molecular orbital features, HOMO (highest occupied molecular orbital) and LUMO (lowest unoccupied molecular orbital), were calculated using quantum mechanics approaches, known as density functional theory (DFT) in material studio.

**FIGURE 2:**
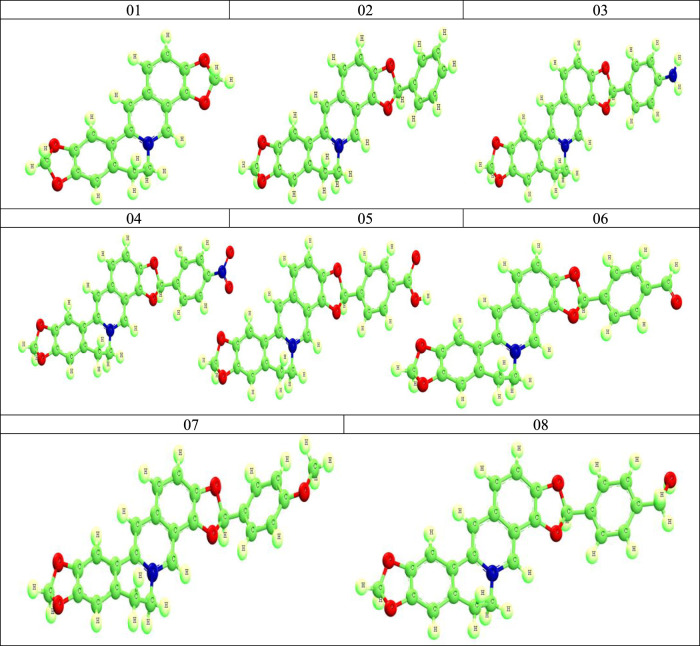
Optimized structures of Coptisine derivatives.

The optimized structures are shown in Figure 2.

### Determination of the data of Lipinski rule

The Lipinski rule of five is helpful for classifying substances into drug-like and non-drug-like groupings. Structural properties such as drug-likeness criteria have been used to more swiftly determine a compound’s drug-like qualities. (Hydrogen bond acceptor, Hydrogen bond donor, TPSA, Bioavailability Index) The key emphasis of the Lipinski five Rule has been generated using Swiss ADME “(http://www.swissadme.ch/index.php)” (Azzam, 2023).

### *In silico* ADMET prediction

The determination of DMPK (drug metabolism and pharmacokinetics) investigation, also known as ADMET (absorption, distribution, metabolism, elimination, and toxicity) experiments, is a crucial component of the drug development process (Ajoy Kumer et al., 2022). Because many drugs cannot reach the final steps due to unfavorable effects and withdrawal from the market. The most reputable and dependable resource for forecasting the AMDET properties is the online database known as pkCSM, which can be found at (“http://biosig.unimelb.edu.au/pkCSM/”) (Pires et al., 2015). ADMET features were finished using this repository and listed in the result and discussion section.

### Preparation of target protein

**FIGURE 3:**
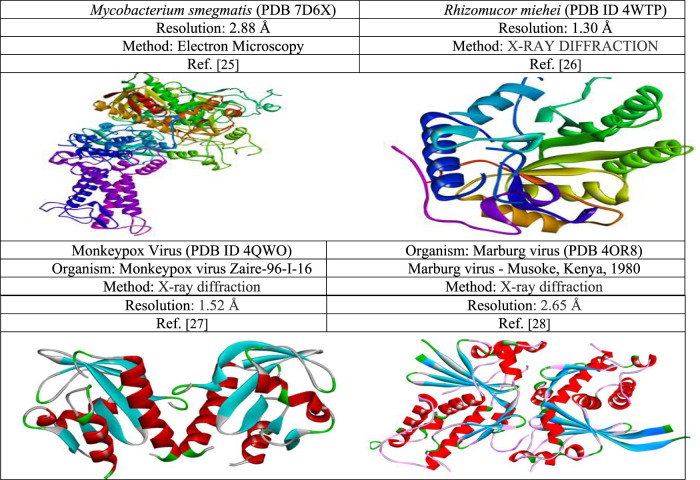
Three-dimensional protein structure of black fungal, Monkeypox, and Marburg virus target proteins used in this study.

The three-dimensional pathogenic fungal proteins *Mycobacterium smegmatis* (PDB 7D6X), *Rhizomucor miehei* (PDB ID 4WTP), and Monkeypox Virus (PDB ID 4QWO) & Marburg virus (PDB 4OR8) were collected from the PDB databank following link https://www.rcsb.org/. Pymol software version PyMolV2.3 scrutinized the protein retrieved from the PDB database (https://pymol.org/2/) (Rosignoli and Paiardini, 2022). All water molecules and unexpected ligands or heteroatoms were eliminated from the protein and preserved as PDB files to obtain the pristine protein. Finally, their energy minimization, and optimization is done with the help of swisspdbviwer (Akash, 2022). The protein information is displayed with a three-dimensional configuration in Figure 3.

### Method for molecular docking

For molecular docking investigation, previously prepared optimized molecules and cleaned protein were uploaded to the PyRx virtual screening application, and run the application in the mode of AutoDock vina (Alam et al., 2016; Pawar and Rohane, 2021). During the docking experiment, each protein was wrapped by different grid box size such as, *Mycolicibacterium smegmatis* (PDB ID 7D6X) center X = 72.897, Y = 74.534, Z = 55.2881-dimension X = 158.417, Y = 155.152, Z = 119.437 and *R. miehei* (PDB ID 4WTP) center X = 48.057, Y = 41.746, Z = 51.503, dimension X = 14.321, Y = 27.735 and Z = 63.678, Monkeypox Virus (PDB ID 4QWO) center X = 12.4697, Y = 15.9818, Z = 16.0634, dimension X = 35.14496, Y = 37.6455, Z = 6.9662, and Marburg virus (PDB 4OR8) center X = 3.0194, Y = −0.7823, Z = 40.2835, dimension X = 38.36585, Y = 58.43047, and Z = 66.85451 When molecular docking was completely done, the docked molecule was loaded to the Pymol software for making them as complex file, and finally discovery studio version 2021 for viewing and analyzing the outcome of protein-ligand binding, Hydrogen bonding, 2d picture of active sites, solvent surface area, ionizability, and aromaticity.

Intermolecular hydrogen bonds between protein and ligand exhibit that 04, 07, and Fluconazole build hydrogen bonds with the residue of the catalytic domain. Fluconazole showed maximum hydrogen bond contact rather than 04 and 07. Eventually, all analyses from the M.D. simulations suggest that 04 and 07 are more stable than Fluconazole and performed a few conformational changes of the protein.

### QSAR and pIC_50_calculation

To establish a statistically valid method for the prediction of the bioactivities of different chemical constituents, quantitative structure-activity relationships (QSARs) have been employed (Roy, 2007). QSAR is a computer modeling technique that reveals correlations between the structural features of bioactive molecules and the biological activities of such substances. The Chemdesk online webtool “http://www.scbdd.com/chemdes/” and the following multiple linear regression (MLR) equation was implemented to calculate the pIC_50_ values. Before calculation the pIC_50_, the MLR equation was developed in excel sheet, and inputted the following data which was obtained from Chemdesk.

pIC_50_ (Activity) = −2.768483965 + 0.133928895 × (Chiv5) + 1.59986423 × (bcutm1) + (−0.02309681) × (MRVSA9) + (−0.002946101) × (MRVSA6) + (0.00671218) × (PEOEVSA5) + (−0.15963415) × (GATSv4) + (0.207949857) × J) + (0.082568569) × (Diametert) (Siddikey et al., 2022).

### Molecular dynamic simulation

The dynamical behavior of nucleic acids, mutant proteins, protein-ligand complexes, and protein-protein interaction is available *via* molecular dynamics (MD) simulation. For finding the stable ligand binding pocket, it is a very handy tool (Ozalp et al., 2018). YASARA dynamics version 21.6.17 was used to run the MD simulation. The hit’s best pose from the virtual screening was chosen, and YASARA Structure’s scene mode was then set up using the default option. The scene mode was put through MD simulations using the YASARA Structure macro’s default parameters for MD run (Prasasty and Istyastono, 2020). Force fields, which use periodic functions for bond rotations, springs for bond lengths and angles, and Coulomb’s equation for ionic interactions to calculate the forces exerted on each atom started from the initial setting. The AMBER14 force field is widely applied for describing macromolecular systems (Ozalp et al., 2018; Siraj et al., 2021). The complex was positioned in the middle of a periodic standard cubic box that also included other atoms and the model. To equalize the system’s charges, Na+ and Cl-ions were inserted to the Transferable Intermolecular Potential3 (TIP3P) water model (Zhang et al., 2020). For energy minimization, every system conducts with the steepest gradient approach (5000cycles). A periodic boundary condition was engaged to play simulations, where the cell size was 10 Å broad than protein size in all events. MD simulations and electrostatic interactions were performed by using particle-mesh Ewald (PME) methods and prescribe some physiological conditions at, 0.9% NaCl, pH 7.4 (Krieger et al., 2006). The setup also used 298 K and one atmosphere, respectively, for temperature and pressure parameters. Finally, 100 ns of MD simulations were executed, and YASARA MACRO’s default script was used to manage further analysis (Shakil, 2021).

## Results and discussion

### Lipinski rule and pharmacokinetics for oral drug

In the initial stages of drug development, the concepts of Pharmacokinetics and Lipinski’s rule give significant assistance that may enhance the possibilities of a biochemical entrance and therapeutic clearance. The predictions of Pharmacokinetics and the drug-likeness properties of medicinal compounds by Swiss ADME (Azzam, 2023). To design a novel medicine for black fungal species infection, Pharmacokinetics and drug-likeness have been investigated comparably to Lipinski’s rule and drug activity utilizing the online source following link https://www.sib.swiss/furnished by the Swiss Institute of Bioinformatics. This rule was established based on the five rules including the molecular weight being less than 500 g/mol, the calculated octanol/water partition coefficient being less than 5 (LogP 5), The number of hydrogen bond donors being less than 5, and the number of hydrogen bond acceptors (particularly N and O atoms) is not more than 10. If the drug-like biomolecules had adhered to this concept, then they should have reflected to use as oral medication. Besides, the topological polar surface area (TPSA) is another useful molecular biomarker in drug discovery and development. It calculates the surface area of a polar or hydrogen-bonding molecule, which might impact solubility, permeability, and other pharmacokinetic features, which anticipate the capability to cross the cell membranes since molecules with high TPSA values may have restricted membrane permeability. Literatures studies reported that the compounds having TPSA values larger than 140 Å^2^ are unlikely to permeate cell membranes, while compounds with TPSA values less than 90 Å^2^ may be able to cross the blood-brain barrier (BBB). Our reported molecules have shown that the TPSA values are less than 90 Å^2^. So, they might cross the BBB, based on this finding (Prasanna and Doerksen, 2009). It is evident from Table 1 that the Lipinski criterion is adhered to by all of the compounds and identified as potential medications. The amount drugs are assimilated into the systemic circulation after it has been administered are called bioavailability. The typical range of bioavailability depend on which routes, the drug is taken by the patient. Our reported molecules have shown the bioavailability score is 0.55 for all compounds, which means 55% of drugs might be present in systemic circulation after administration (Chen et al., 2022).

**TABLE 1:** Summary of ligands calculated results for Lipinski rule, pharmacokinetics and drug likeness activities.

Ligand No	Molecular weight	Number of rotatable bonds	Hydrogen bond acceptor	Hydrogen bond donor	Topological polar surface area (Å^2^)	Consensus Log*P*_o/w_	Lipinski rule		Bioavailability score
							Result	Violation	
01	320.32	00	04	00	40.80	2.40	Yes	00	0.55
02	396.41	01	04	00	40.80	3.57	Yes	00	0.55
03	411.43	01	04	01	66.43	3.07	Yes	00	0.55
04	441.46	02	04	02	86.62	3.00	Yes	00	0.55
05	440.41	02	06	01	78.10	3.18	Yes	00	0.55
06	424.42	02	05	00	57.87	3.31	Yes	00	0.55
07	426.24	02	05	00	50.03	3.61	Yes	00	0.55
08	426.44	02	05	01	61.03	3.15	Yes	00	0.55

### Chemical descriptor (HOMO-LUMO) calculation

HOMO stands for the highest occupied molecular orbital, while LUMO stands for the lowest unoccupied molecular orbital. The molecular orbitals (HOMO-LUMO) and chemical reactivity descriptors that are conceived by the computer program, and they are mathematical representations of the different properties which present in the chemical structures (Alam et al., 2018). These estimations of chemical properties are obtained by utilizing the B3LYP functional to material studio, and it is mostly related to HOMO-LUMO energy gap. The optimal HOMO-LUMO energy gap for organic compounds is between −6.00 and −9.00 eV and is acknowledged as perfectly fitting for organic molecules (Nath et al., 2021). The energy gaps of molecular orbitals are explored to evaluate the electrical conduction capabilities of atoms and molecules. Good physical and chemical stability can be maintained through the use of energy gaps. The high chemical stability must be depended on the size of the HOMO-LUMO gap (Kumer et al., 2019). Compounds with a small energy gap have a greater atomic system but lower chemical stability due to HOMO-LUMO being close to each other. According to this research, the E-gap spans from 8.831 eV to 8.864 eV. This shows a large E-gap, which indicates that the molecules in consideration have better chemical stability and a lower atomic system. The chemical potential, hardness, and softness are all valuable parameters for determining the therapeutic potential of biologically active molecules. Usually, the softness of drugs like molecules should be lower than the hardness of the drug. The absorption rate is proportional to the lower softness, and the hardness must be at around 4.000 kcal/mol for optimal biological flexibility (Akter and Bhuiyan, 2022). Here, softness ranges from 0.2256 to 0.2265, and hardness ranges from 4.415 to 4.4427. So, in our current investigation, all the coptisine derivatives have greater hardness and softness scores than the standard; these mentioned drugs may take longer to break down in the physiological system. The chemical potentiality varies from a minimum of 5.4485 eV to a high of 5.5925 eV, with all these values clustering close to the standard value of 8.831 eV (See Table 2). Finally, it is concluded that all derivatives of coptisine are potential and better stable according to this finding.

**TABLE 2:** Chemical reactivity descriptor analysis.

S/N	I = − HOMO	A = -LUMO	Energy gap E (gap) = I-A (eV)	Chemical potential $\left(\mu \right)=\frac{I+A}{2}$	Hardness $\left(\eta \right)=\frac{I-A}{2}$	Softness $\left(\sigma \right)=\frac{1}{\eta }$
01	−9.886	−1.022	8.864	5.454	4.432	0.2256
02	−9.883	−1.021	8.862	5.452	4.431	0.2257
03	−9.878	−1.019	8.859	5.4485	4.429	0.2258
04	−10.008	−1.177	8.831	5.5925	4.415	0.2265
05	−9.993	−1.139	8.854	5.566	4.427	0.2259
06	−9.995	−1.141	8.852	5.568	4.427	0.2259
07	−9.974	−1.129	8.845	5.5515	4.422	0.2261
08	−9.979	−1.134	8.845	5.5565	4.423	0.2261

### Molecular docking analysis against black fungus

Docking is considered one of the most promising techniques in structure-based drug design (Fischer et al., 2021). This technique calculates the preferred orientation of a compound and when it is attached to protein with ligands to develop a stable combination (El-Demerdash et al., 2021; Ouassaf et al., 2021). Docking may also show the signatory of small molecule ligand on appropriate target region during the formation of the drug-protein complex. H-bonding and hydrophobic bonding are the essential factors in docking values because they perform a substantial role in structurally based medication development (Cichero et al., 2021). The substance is categorized as a standard medication when the docking score exceeds −6. 000 kcal/mol (Rahman et al., 2022a; Kumer et al., 2022b). In this investigation, the coptisine derivatives show outstanding binding affinities against both species of black fungus. Specifically, the binding affinities range from −9.4 kcal/mol to −11.0 kcal/mol against *R. miehei* (PDB ID 4WTP). Once a significantly improved binding affinity has been achieved compared to *R. miehei,* another species of black fungus was picked up (*Mycolicibacterium smegmatis*) and performed molecular docking experiment. Then, it is observed that the binding affinity is about −10.4 kcal/mol to −12.8 kcal/mol. In each condition, Fluconazole is used as a standard drug, and in comparison, newly developed coptisine derivatives have achieved better binding energy mentioned in Table 3 **.** So, these reported molecules could be used as potential inhibitors for treating black fungal species infection.

**TABLE 3:** Binding affinities of docked ligand calculated against Black Fungus.

Drug molecules No	*Rhizomucor miehei* (PDB ID 4WTP)	*Mycolicibacterium smegmatis* (PDB ID 7D6X)
	Binding Affinity (kcal/mol)	Binding Affinity (kcal/mol)
01	−9.4	−10.4
02	−11.0	−11.7
03	−10.5	−10.7
04	−10.4	−12.8
05	−10.7	−11.1
06	−10.4	−10.8
07	−10.5	−12.2
08	−10.1	−11.0
Fluconazole	−7.0	−8.0

### Binding affinities analysis against pathogenic Monkeypox and Marburg virus

Historically and literature findings reported that coptisine has a broad spectrum of pharmacological effects such as antidiabetic, antimicrobial, antiviral, anti-cancer (Wang et al., 2017). So, based on the literature studies and literature, Monkeypox, and Marburg virus are also included in this research to analyze what types of activities presented the synthetic derivatives of coptisine against the Monkeypox and Marburg virus. So, the molecular docking was conducted, and it was reported that the binding affinities against Monkeypox virus (PDB ID 4QWO), −8.3 kcal/mol, −8.5 kcal/mol, −9.3 kcal/mol, −10.8 kcal/mol, −9.5 kcal/mol, −9.8 kcal/mol, and −9.2 kcal/mol in ligands (02–08). Besides that, the binding affinities ranges against Marburg virus (PDB 4OR8) is −8.3 kcal/mol to −8.7 kcal/mol for ligands (01–08). As there is no medication against Monkeypox and Marburg virus, we have considered an established antiviral and performed with these two pathogens to compare with our studies drugs. Overall findings against Monkeypox and Marburg virus are determined as outstanding affinities compared to standard (acyclovir) showing in Table 4 **.** So, they might be suggested as potential drugs for inhibiting Monkeypox and Marburg virus disease.

**TABLE 4:** Binding affinities of docked ligand calculated against Monkeypox and Marburg virus.

Drug molecules No	Monkeypox virus (PDB ID 4QWO)	Marburg virus (PDB 4OR8)
	Binding Affinity (kcal/mol)	Binding Affinity (kcal/mol)
01	−8.3	−8.5
02	−8.5	−8.7
03	−9.3	−8.3
04	−9.0	−8.6
05	−10.8	−8.5
06	−9.5	−8.3
07	−9.8	−8.2
08	−9.2	−8.3
Standard (Acyclovir)	−6.3	−5.8

### Protein-ligand interaction and molecular docking pose

**FIGURE 4:**
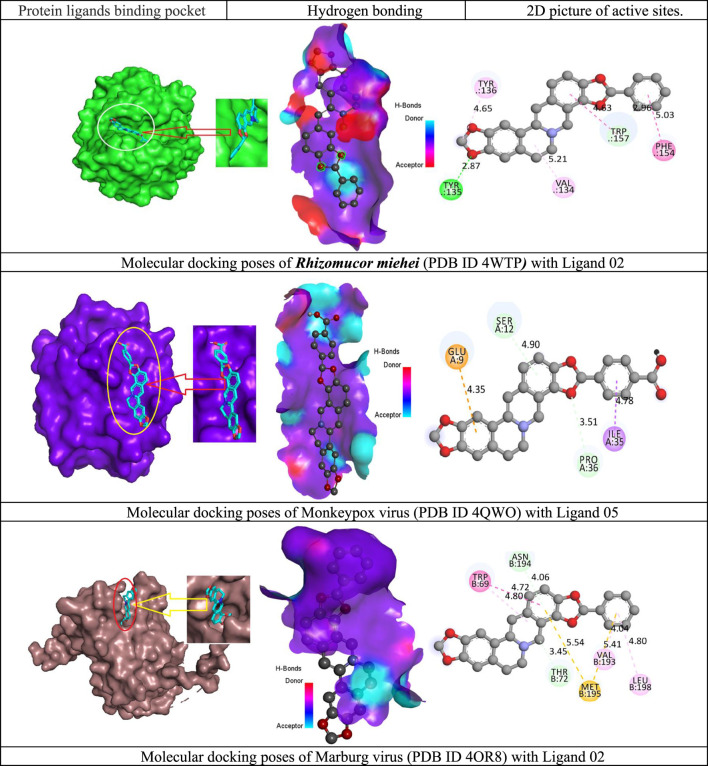
Docking interactions between the proposed compound and targeted pathogen.

The ligand-protein interaction is essential when developing a novel medicine since it reveals how well a drug will react to the protein of a black fungus or any targeted binding receptor. The interaction of the new drug candidate with the *R. miehei* (PDB ID 4WTP), *Mycolicibacterium smegmatis* (PDB ID 7D6X) of black fungus, and Monkeypox and Marburg virus have been explored with 2d active residue and hydrogen bonding system. Figure 4 demonstrates that there are different sorts of bonds, especially H-bond and hydrophobic bonds are denoted in most cases. However, the Van der Waal bond is not prevalent for all medications. The active is prediction TYR:135, VAL: 134, PHE:154, TRP:157, TYR:136, in ligand 02 against *R. miehei,* LEU A:432, GLU A:413, ARG A:423, HIS A:46, ALA A:50 against *Mycolicibacterium smegmatis* in ligand 07, similarly, SER A:112, GLU A:109, PRO A:36 against Monkeypox virus in ligand 05, and TRP B:69. ASN B: 194, THR B:72, MET B: 195, VAL B:193, LEU B: 198 against Marburg virus in ligand 02 mentioned in Figure 4.

### Ionizability and aromaticity analysis

#### Ionizability

Ionization is a term used in science and chemistry to describe any mechanism by which electrostatic interactions particles or atoms may be switched to electrically charged particles or atoms (ions) while receiving or shedding electrons (Aleksandrov et al., 2010). The digestive system’s epithelial cells are responsible for keeping the stomach healthy. To get into the bloodstream, a medication must penetrate through it or penetrate endothelial tissues. The cell membranes of certain medicines may operate as a barrier. Semi-permeable membranes are made up of phospholipid bilayers. Extremely tiny, uncharged substances may penetrate pristine lipid bilayers. Because ionic compounds are electrified, the assimilation of a component will be affected whether it is discharged or not. While molecules with positive charges have a higher solubility, those with negative charges have higher permeability. Exchange proteins and channels let certain chemicals pass from the lumen into the bloodstream more efficiently (Mannens et al., 2002).

**FIGURE 5:**
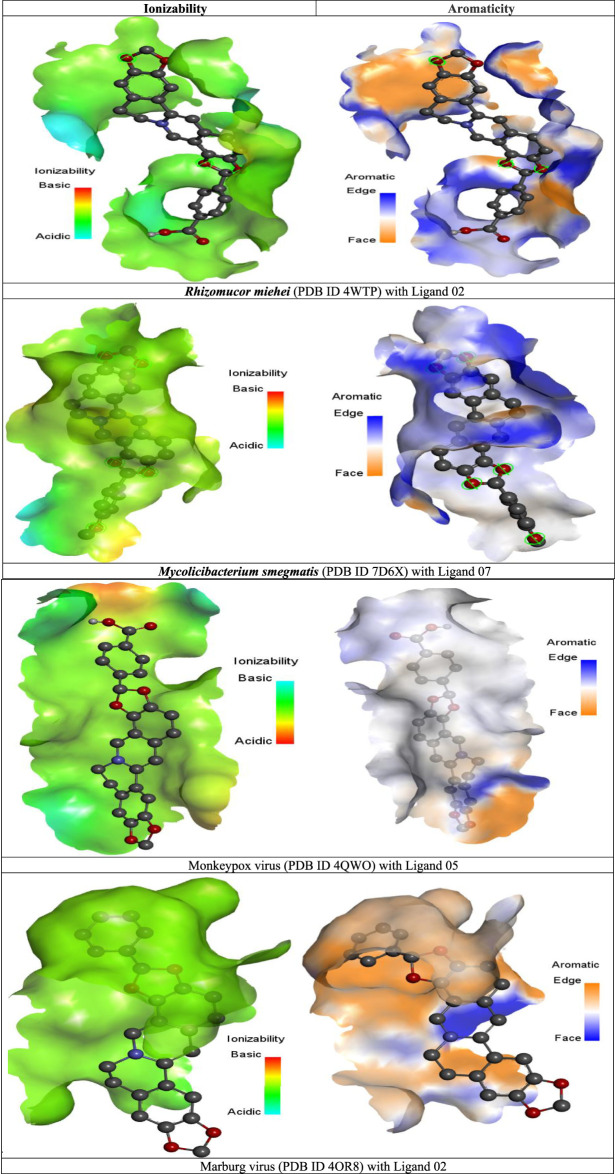
Graphical illustration of ionizability, and aromaticity analysis.

In the illustration of ionizability, the red color represents acidic, the sky blue is regarded as basic, and the green color indicates neutral or slightly acidic or basic (Figure 5). But our investigation suggested that almost all the molecules have a greater possibility of neutralizing, which regards, they could rapidly penetrate semi-permeable membranes and reach the bloodstream or systemic circulation.

#### Aromaticity

In order to produce drugs reasonably, one must be able to anticipate and optimize the non-covalent interactions between organic ligands and protein (Brylinski, 2018). The aromatic arrangement has indeed been established for a long time as one of the primary factors of ligand-protein integrations that are responsible for maintaining–chemical bonding (π-π) (Kar et al., 2013). Based on the illustration of aromaticity, it can be seen that the edge and face of the engagement among pharmaceuticals acting as ligand and the protein of the black fungus, together with the pocket, demonstrate how the ligand has coupled with the peptide and where it has generated a bonding.

#### Molecular dynamic simulation

**FIGURE 6:**
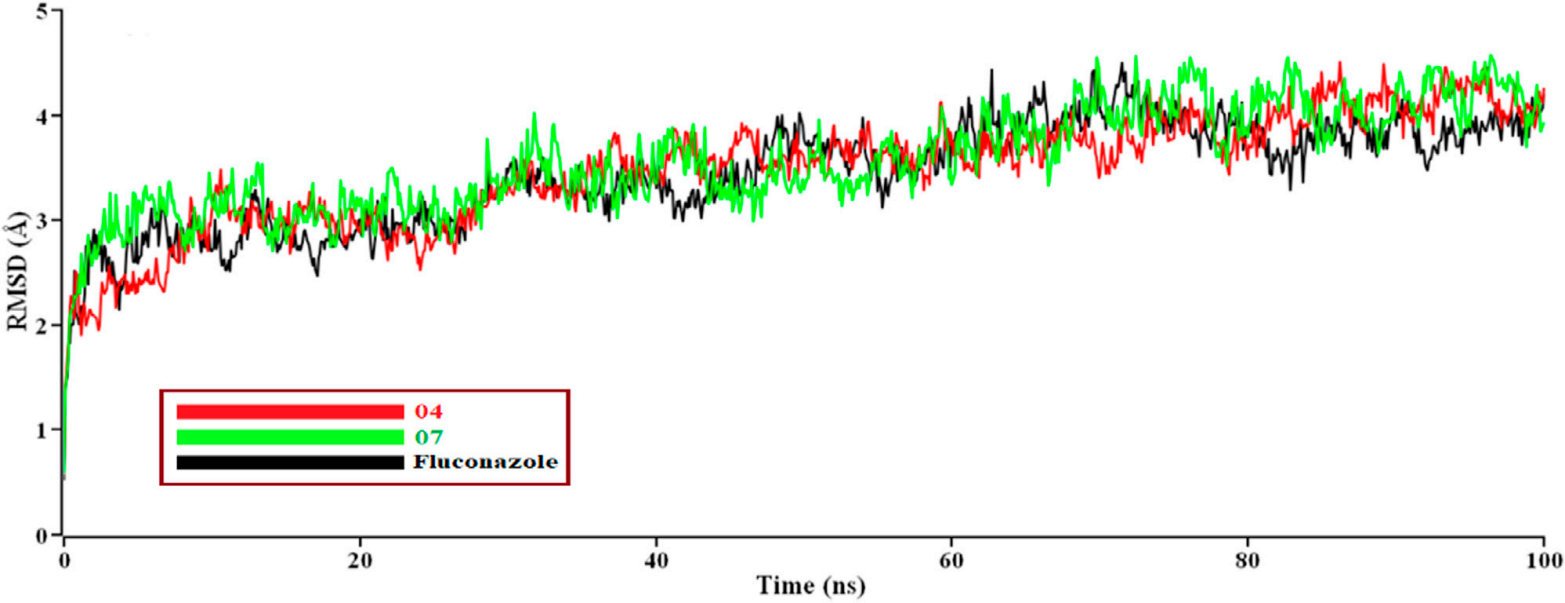
The time series of the RMSD of Cα atoms for protein *Mycobacterium smegmatis* (PDB ID 7D6X) and ligand. Here, red and green lines denote the 04 and 07 complexes, and the black lines denote the subjected drug Fluconazole, respectively.

For understanding the nature of structural stability and flexibility, some selected compounds (04, 07, and fluconazole) have performed MD simulation (Based on maximum docking score with proteins); here, Fluconazole has been used as a standard reference medication, and we performed M.D. simulations for 100 ns. RMSDs of the Cα atoms for protein and ligand were calculated and plotted time-dependent (Figure 6) (Junaid et al., 2019)**.**

Evaluate protein behavior during M.D. simulation; as seen in the plot, 07 complexes showed high fluctuation in RMSD at 95 ns–97 ns, indicating complex are not perfectly stable as complex 04 and Fluconazole. This point indicates that at 95 nano second to 97 nano second, the RMSD of complex (07) showed high fluctuation, which means lower stability compare to complex 04 and Fluconazole, or deviation from the complex 04 and Fluconazole. So, at 95 nano second to 97 nano second, the complex 07 has shown lower stability. Other protein-ligand complex results were closer to the 07 value also obtained in the graph for better results on how 04, 07, and Fluconazole influence the binding mode with *Mycobacterium* smegmatis (PDB ID 7D6X). The structural change of the three complexes was tested through the root mean square fluctuation (RMSF), the radius of gyration, and the solvent-accessible surface area of the protein-ligand complex (Figure 6).

**FIGURE 7:**
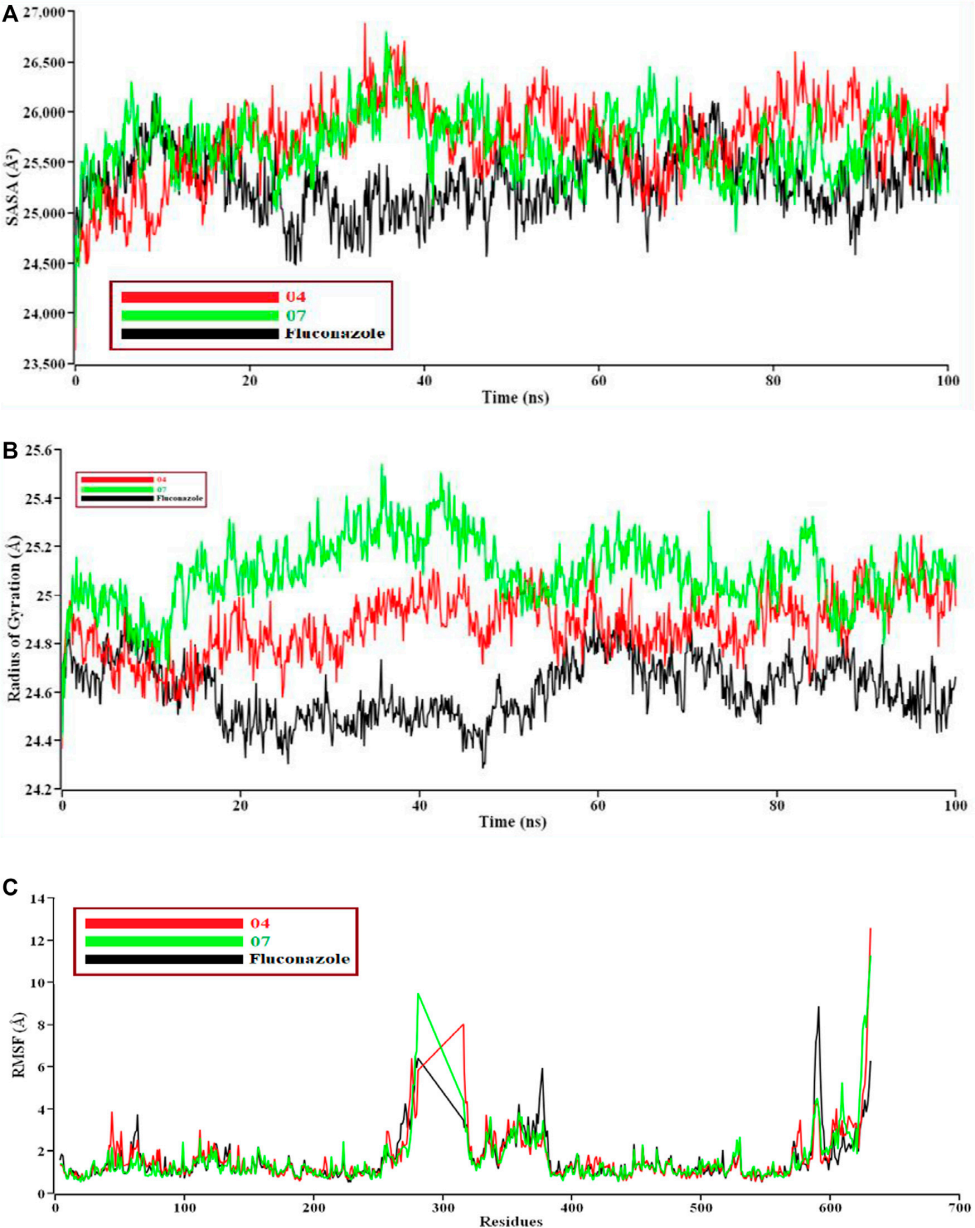
The structural behavior changes of protein employing a) solvent accessible surface area (SASA), 8b) radius of gyration, and 8c) root means square fluctuations (RMSF) analysis. Here, the red line indicates 04, and the green and black lines indicate 07 and the Fluconazole complex, respectively.

**FIGURE 8:**
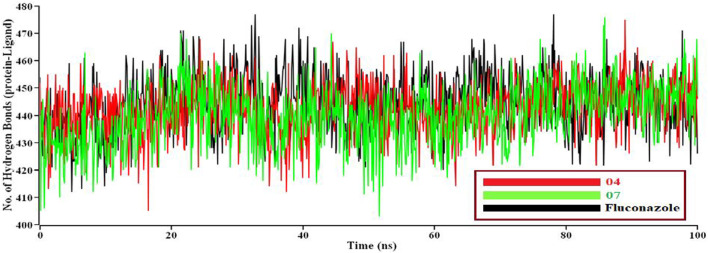
A total number of hydrogen bonds formed between protein-ligand complexes during M.D. simulations. Here, the red line indicates 04 complexes, and the green and black lines indicate 07 and the Fluconazole complex, respectively.

If solvent enters into the binding site, the pocket can be destroyed. We need protein-ligand tight interaction. (Figure 7A). represents 04 compounds showed high SASA value after 33 ns of simulation, it may not reduce the protein expansion. In contrast, (Figure 7B), demonstrates the radius of gyration value; 07 compounds showed high value than 04 and Fluconazole, denoting loose packing of protein structure. RMSF value (Figure 7C) reflects the flexibility of the whole residue in the protein. High fluctuations were performed in some positions, resulting in better results, ranging from 631–635 residues, including the 04 and 07 complexes. Finally, the hydrogen bond interaction formed within protein and ligand showed in Figure 8.

### QSAR an pIC_50_ analysis of coptisine derivatives

The trustworthy *in silico* approach known as quantitative structure-activity relationship (QSAR) was established to simulate the bioactivity utilizing the chemical structure. Before the exact chemical synthesis begins, it is possible to make predictions about the bioactivity of the prospective bioactive compounds. The projection is built based on the structural characteristics involved in bioactivity. Through the use of QSAR models, the structural and molecular features may be expressed (Ahmad Pasha et al., 2010).

The mathematical QSAR model working of multiple linear regression which had been built by analyzing the computational IC50 values similar to pIC50 [-log (IC50)]. From the ChEMBL open-source website (Gaulton et al., 2012). ChEMBL was developed by more than a million bioactive molecules and was founded from the eight most approved biological characteristics, including hiv5, bcutm1, MRVSA9, MRVSA6, PEOEVSA5, GATSv4, J, and diameter, among others. Moreover, the IC_50_ values are closely correlated to its structural chain, and this value changes with the modification in its side chain. The score of IC_50_ increases as the molecular weights of the medicine increases, but it must remain under 10.00 in order to be considered an efficient medication. Mentioned the Table 5, it has been reported that the pIC_**50**_ value is reported as 4.87–5.45, which falls in acceptable ranges and could be said to be potential drug (Rahman et al., 2022b). So, the pIC50 value of drugs (01–08) should be an efficient drug since the value is not more than 10.0.

**TABLE 5:** Data of QSAR and pIC_50_.

Ligand	Chiv5	bcutm1	MRVSA9	MRVSA6	PEOEVSA5	GATSv4	J	Diametert	pIC_50_
1	2.852	4.121	10.772	42.092	00	0.839	1.24	11	4.87
2	3.476	4.127	10.772	77.987	30.332	1.103	1.055	15	5.31
3	3.556	4.128	16.46	71.921	00	1.086	1.039	16	5.08
4	3.572	4.128	16.46	82.035	00	1.033	1.009	17	5.14
5	3.593	4.128	16.772	87.485	12.33	1.037	1.009	17	5.20
6	3.576	4.128	17.059	77.485	24.65	1.063	1.022	17	5.30
7	3.572	4.128	10.772	71.921	00	0.996	1.022	17	5.31
8	3.614	4.128	10.772	77.485	24.265	1.063	1.022	17	5.45

### *In silico* ADMET data prediction

Drug metabolism and pharmacokinetics (DMPK) studies are a key component of the drug development process. Investigations like this popularly stand for ADMET (Absorption, Distribution, Metabolism, and Toxicity) because they explore how medications are metabolized and removed from the physiological system. These experiments contribute to the process of assessing the efficacy of a potential new drug. For instance, the absorption, or the amount of drug and how quickly it is absorbed into the body, distribution refers to how the drug is distributed within the body and how fast and broadly it has been supplied. The term “metabolism” refers to the pace at which a drug is broken down and its mechanism of action; the metabolites form it generates, the elimination, has been defined as outlining how and how quickly the medicine departs the body. Finally, the toxicity-has been described. Whether or not it is beneficial or harmful to human use (Selick et al., 2002; Chalkha et al., 2022).

The ADMET profile of the medicine, as determined by a search of an online database called pkCSM and displayed in Table 6 for the purpose of computational prediction. The result reported that the aqueous solubility ranges −2.948 to −4.453, which means they are moderate to highly soluble (Tallei et al., 2022); the Caco-2 Permeability ranges were found to be 0.654–1.095, while all of the drug candidates have a quick absorption rate in the human digestive tract, which is indicated by a range of values between (96.972%–100%). Besides, around 08 out of 07 drug candidates may inhibit by CYP450 1A2 Inhibitor, and none of them can be Substrate by CYP450 2C9. The Total Clearance 0.874 mL/min/kg–1.389 mL/min/kg and Max. tolerated dose 0.449 log mg/kg/day while the minimum tolerated dose −0.21 log mg/kg/day. Finally, they all are free from skin sensitization and hepatotoxicity. The overall ADMET, and Pharmacokinetics properties is satisfied to be potential medication. These drug candidates are suggested to explored further laboratory experiment such as synthesis, and *in vitro* or *in vivo* experiment.

**TABLE 6:** Summary of calculation of ADMET results for selected 08 compounds.

S/N	Absorption			Distribution	Metabolism		Excretion		Toxicity		
	Water solubility Log S	Caco-2 Permeability x 10^–6^	Human Intestinal Absorption (%)	VDss (human)	CYP450 1A2 Inhibitor	CYP450 2C9 Substrate	Total Clearance (mL/min/kg)	Renal OCT2 substrate	Max. tolerated the dose log mg/kg/day	Skin Sensitization	Hepatotoxicity
01	−2.948	1.195	99.223	0.755	Yes	No	1.287	Yes	−0.21	No	No
02	−3.22	1.019	100	−0.96	Yes	No	1.217	No	0.57	No	No
03	−3.979	0.989	96.972	0.20	Yes	No	1.378	No	0.307	No	No
04	−4.187	0.977	98.11	0.012	No	No	1.034	No	0.181	No	No
05	−3.562	0.974	97.21	−0.331	Yes	No	0.874	No	0.449	No	No
06	−4.453	0.654	99.971	0.189	Yes	No	1.257	No	0.311	No	No
07	−3.831	0.692	99.90	0.018	Yes	No	1.291	No	0.401	No	No
08	−4.043	0.643	97.701	0.181	Yes	No	1.389	No	0.325	No	No

## Conclusion

Currently, there is a limited number of drugs available against pathogenic black fungus (*R. miehei* and *Mycolicibacterium smegmatis*), Monkeypox, and Marburg virus. So, in this innovative and advanced *in silico* investigation, various *in silico* approaches are applied to find potential inhibitors against two species of pathogenic black fungus (Rhizomucor miehei and Mycolicibacterium smegmatis), Monkeypox, and Marburg virus by modification of coptisine with different molecular modeling approaches. Firstly, design the coptisine derivatives by structural modification. Then, the molecular docking, drug-likeness, ADMET, Molecular dynamic simulation, and QSAR, etc., are gradually conducted. The highest docking score reported for a range such as this is 9.4 kcal/mol to 11.0 kcal/mol, and it is for *R. miehei* (PDB ID 4WTP). Furthermore, it has an over the potential of −10.4 kcal/mol to 12.8 kcal/mol for *Mycobacterium smegmatis* (PDB ID 7D6X). Similarly, the maximum affinities against Monkeypox virus (PDB ID 4QWO), −8.3 kcal/mol, −8.5 kcal/mol, −9.3 kcal/mol, −10.8 kcal/mol, −9.5 kcal/mol, −9.8 kcal/mol, and −9.2 kcal/mol in ligands (02–08). Besides that, the binding affinities ranges against Marburg virus (PDB 4OR8) is −8.3 kcal/mol to −8.7 kcal/mol for ligands (01–08).Correspondingly, compared to FDA-approved standard Fluconazole and standard (Acyclovir). It is acceptable to conclude that the evaluated bioactive coptisine derivatives have significantly superior binding affinity and dynamic molecular accounting to indicate their stability. Besides, the quantum calculation HOMO-LUMO gap is also acceptable ranges. After completing the comprehensive study, it was observed that all of the medication candidates exhibited the following characteristics: better solubility in water, absence of any toxic effect; high gastrointestinal (G.I) absorption rate; fulfillment of the Lipinski rule; and drug-like aspects. Therefore, these mentioned drug candidates have been determined to be the effective medication for inhibition of the deadly black fungus pathogen, Monkeypox, and Marburg virus presumably, if these pharmaceuticals are put in clinical trial or laboratory investigation, they will be substantially fewer negative impacts than those of established medications according to our computational data.

## Data Availability

The original contributions presented in the study are included in the article/supplementary material, further inquiries can be directed to the corresponding authors.
